# Amélioration de la calcinose tumorale de la main droite après para thyroïdectomie chez un hémodialysé chronique

**DOI:** 10.11604/pamj.2016.24.30.8814

**Published:** 2016-05-09

**Authors:** Jaouad El Maghraoui, Mohamed Hammou, Nadia Kabbali, Mohamed Arrayhani, Tariq Sqalli Houssaini

**Affiliations:** 1 Service de Néphrologie, CHU Hassan II, Equipe de Recherche REIN, Faculté de Médecine et de Pharmacie, Fès, Maroc; 2 Service d'Hémodialyse, Hôpital Ibn Lkhatib, Fès, Maroc

**Keywords:** Patient on chronic hemodialysis, hyperparathyroidism, calcinosis, parathyroidectomy, Patient on chronic hemodialysis, hyperparathyroidism, calcinosis, parathyroidectomy

## Abstract

Les calcifications des tissus péri articulaires sont fréquentes chez les insuffisants rénaux chroniques en hémodialyse. Nous rapportons le cas d'un hémodialysé chronique depuis 10 ans qui a présenté une calcinose pseudo tumorale isolé à la main droite nettement améliorée après para thyroïdectomie. A travers cette observation, nous montrons l'impact du para thyroïdectomie sur la calcinose pseudo tumorale.

## Abstract (en)

Periarticular tissue calcifications are common in patients with chronic renal failure undergoing hemodialysis. We report the case of a patient on chronic hemodialysis for 10 years with significant improvement of isolated pseudotumoral calcinosis of the right hand after parathyroidectomy The aim of this study was to show the impact of parathyroidectomy on pseudotumoral calcinosis.

## Introduction

Les calcifications des tissus péri articulaires sont fréquentes chez les insuffisants rénaux chroniques en hémodialyse. L'hyperparathyroïdie secondaire est l'une des principales complications chez l'HDC et peut conduire à une calcinose [1, 2] qui prend dans de rares cas une formes massive, pseudo-tumorale, de physiopathologie non univoque et de traitement difficile. Malgré les progrès des traitements médicaux (analogues de la vitamine D actif par voie orale, calcium par voie orale…) la parathyroidectimie permet la prévention de l'hypercalcémie chronique, la résorption osseuse et la calcinose [3, 4] en particulier chez les patients atteint d'une hyperplasie de la parathyroïde qui présente des troubles métaboliques réfractaires au traitement médicale [1, 5]. Nous rapportons un cas d'une calcinose pseudo-tumorale localisée à la main droite chez un hémodialysé chronique améliorée de façon spectaculaire après para thyroïdectomie.

## Patient et observation

**Figure 1:**
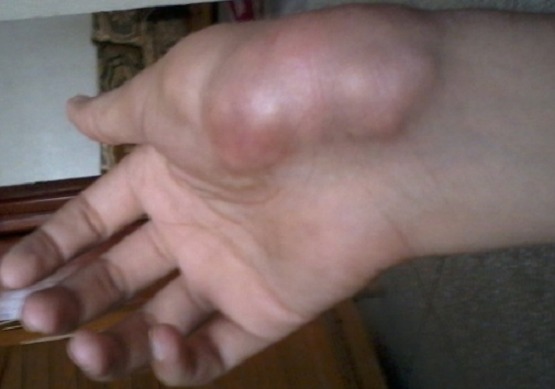
Calcinose pseudotumorale de la main droite avant parathyroïdectomie

**Figure 2:**
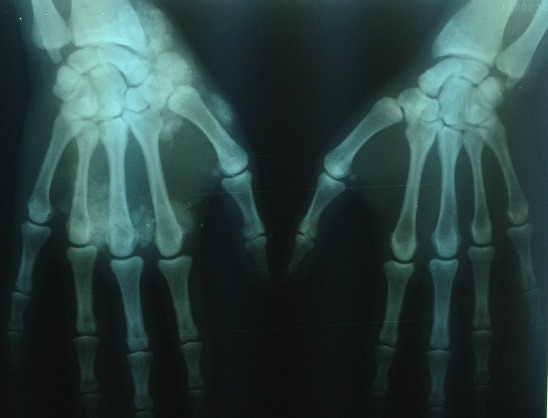
Radiographie standard des deux mains objectivant une calcinose pseudotumorale de la main droite avant par thyroïdectomie

**Figure 3:**
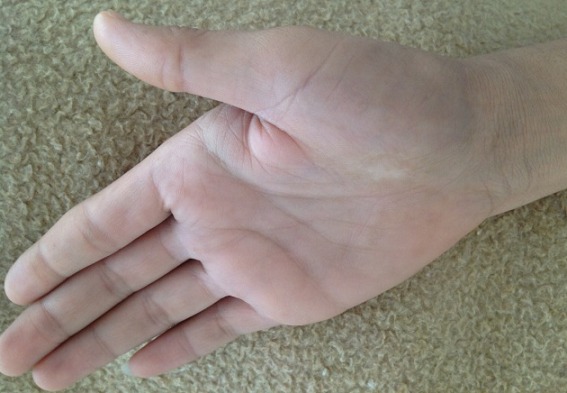
Régression de la calcinose pseudo tumorale de la main droite quatre mois après para thyroïdectomie

**Figure 4:**
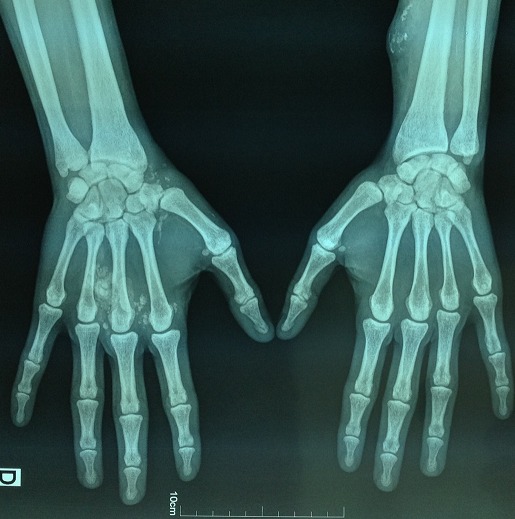
Radiographie standard objectivant la régression de la calcinose pseudo tumorale de la main droite quatre mois après para thyroïdectomie

Il s'agit d'un patient âgé de 32 ans, hémodialysé chronique sur néphropathie indéterminée depuis 10 ans, ayant un antécédent de surdité isolée du coté gauche depuis l'enfance non explorée sans contexte familial d'hématurie, ni surdité ou d'insuffisance rénale. Le patient a été traité également il y'a deux ans pour une tuberculose pleurale avec bonne évolution. Depuis un an et demi, le patient se plaint de douleurs d'allure inflammatoire au niveau du poignet et l'articulation métacarpo-phalangiennes de la main droite. Six mois plus tard nous avons noté l'apparition de deux masses; une au niveau de la face palmaire mesurant 10 cm au niveau de la loge hypothenarienne et l'autre au niveau de la face dorsale de la main mesurant 14cm en regard des articulations métacarpo-phalangiennes (Figure 1). Le bilan biologique a mis en évidence une calcémie normale à 96 mg/l, une hyperphosphatémie à 49 mg/l, et une hyperparathyroïdie avec un taux de PTH 1-84 à 1363 pg/ml, sous traitement médical à base de calcium par voie orale et vitamine D3. La radiographie standard des deux mains a objectivé une calcinose pseudo-tumorale péri-articulaire de la main droite (Figure 2). Par ailleurs l’échographie cervicale a objectivé un nodule parathyroïdien gauche mesurant 6 mm, d'aspect tissulaire homogène et bien limité, sans anomalie parathyroïdienne droite, complétée par une scintigraphie au MIBI objectivant une fixation au niveau du tissu parathyroïdien inférieur gauche. Devant la persistance de l'hyperparathyroïdie secondaire malgré le traitement médical et vu l'apparition récente de la calcinose pseudo-tumorale péri-articulaire, l'indication d'une para thyroïdectomie s'est avérée nécessaire. Le patient a bénéficié d'une résection 7/8 avec conservation de la moitié de la parathyroïde supérieure droite. L’évolution a été marquée par la baisse du taux de PTH 1-84 à 41,5 pg/ml à un mois et à 70,2 pg/ml après quatre mois de suivi, avec une calcémie et une phosphatémie normales (Tableau 1). Nous avons noté aussi une bonne évolution clinique et radiologique, avec régression importante des deux masses de la main droite (Figure 3, Figure 4). A un an de suivi, le patient ne présente plus de douleurs articulaires avec toujours une régression des deux masses et un bilan phospho-calcique satisfaisant.

**Tableau 1:** Bilan phospho-calcique avant et après para thyroïdectomie

	Avant para thyroïdectomie	Un mois après para thyroïdectomie	Quatre mois après parathyroïdectomie
**calcium**	96 mg/l	101 mg/l	92 mg/l
**phosphore**	49 mg/l	40 mg/l	42 mg/l
**PTH 1-84**	1363 pg/ml	41,5 pg/ml	70,2 pg/ml

## Discussion

L′hyperparathyroïdie secondaire est l′une des complications fréquentes difficiles à gérer chez les hémodialysés chroniques. La calcinose pseudo-tumorale est caractérisée par le développement de masses calcifiées dans les parties molles des grosses articulations. Elle peut constituer une complication de l'insuffisance rénale chronique. La physiopathologie est mal connue. Certains auteurs évoquent le rôle de l'augmentation du rapport sérique Ca/P, ainsi que du phosphore. L'hyperparathyroïdie secondaire est également incriminée [6]. Le traitement de la calcinose doit cibler les principaux facteurs précipitants, notamment, l'hyperparathyroïdie secondaire, l'hypocalcémie et l'hyperphosphatémie. Plusieurs rapports de cas ont indiqué que l′hyperparathyroïdie secondaire et la calcinose peuvent être complètement ou remarquablement améliorée après para thyroïdectomie [7, 8], comme le cas de notre patient qui a nettement amélioré sa symptomatologie après para thyroïdectomie. Etant donné que le patient avait également une hyperparathyroïdie, les auteurs ont émis l′hypothèse que les lésions cutanées ont été causées par une sensibilisation à l′hormone parathyroïdienne (PTH). Autres rapports de cas précoces de calciphylaxie également spéculé que l′hypersensibilité à la PTH a joué un rôle majeur dans le développement des lésions cutanées [9]. Le traitement standard pour hyperparathyroïdie secondaire; adénome ou hyperplasie est la résection chirurgicale. La para thyroïdectomie peut actuellement être effectuée en toute sécurité et efficacement, Même si elle est envahissante. La para thyroïdectomie mini-invasive utilisant de plus petites incisions a été récemment réalisée, et d′autres méthodes, telles que l′injection d′éthanol sont également utilisé dans la thérapie clinique [10, 11].

## Conclusion

La calcinose tumorale est une complication rare de l'hémodialyse, outre la correction des facteurs favorisants (élévation du produit CaxP, HPT sévère, remodelage osseux bas), l'utilisation d'un bain de dialyse pauvre en calcium peut être une thérapeutique efficace et bien tolérée. Sa durée doit être limitée à cause de l'aggravation de l'hyperparathyroïdie et du risque associé de calcifications vasculaires.
